# Traditional Chinese Medicine Aconiti Radix Cocta Improves Rheumatoid Arthritis via Suppressing COX-1 and COX-2

**DOI:** 10.1155/2021/5523870

**Published:** 2021-09-07

**Authors:** Shuang Li, Rui Li, Ya-Xin Xu, Jan P. A. Baak, Ji-Hai Gao, Jian-Qin Shu, Li-Jia Jing, Xian-Li Meng, Chuan-Biao Wen, Yan-Xiong Gan, Shi-Chao Zheng, Yong Zeng

**Affiliations:** ^1^School of Pharmacy, Chengdu University of Traditional Chinese Medicine, Chengdu 611137, China; ^2^School of Medical Information Engineering, Chengdu University of Traditional Chinese Medicine, Chengdu 611137, China; ^3^Department of Pathology, and Department of Research,, Stavanger University Hospital, Dr. Jan Baak AS, Risavegen 66, Stavanger 4056, Norway

## Abstract

According to Traditional Chinese Medicine (TCM), Aconiti Radix Cocta (AC) is clinically employed to expel wind, remove dampness, and relieve pain. We evaluated the antirheumatoid arthritis (RA) activities and underlying mechanisms of AC. The chemical constituents of AC were analyzed by high-performance liquid chromatography (HPLC) using three reference compounds (benzoylaconitine, benzoylmesaconine, and benzoylhypacoitine). The anti-RA effects of AC were evaluated in adjuvant-induced arthritis (AIA) rats by hind paw volume and histopathological analysis. The effects of AC on inflammatory cytokines (IL-1*β* and IL-17A) were determined by enzyme-linked immunosorbent assay. The regulation of cyclooxygenases (COX-1 and/or COX-2) was determined by Western blot and real-time quantitative reverse transcription polymerase chain reaction analyses. AC significantly reduced paw swelling, attenuated the inflammation and bone destruction in joint tissues, and reduced IL-1*β* and IL-17A in the serum. Moreover, AC downregulated the expression of COX-1 and COX-2 in the synovial tissues. We also identified that AC possesses significant anti-RA activities on AIA, which may be ascribed to the regulation of inflammatory cytokines IL-1*β* and IL-17, as well as to the inhibition of arachidonic acid signaling pathways. Our findings provide theoretical support for AC as an effective nature-derived therapeutic agent for RA treatment.

## 1. Introduction

Rheumatoid arthritis (RA) is a polyarticular (often but always) symmetric disease that involves multiple joints bilaterally, characterized by progressive joint destruction and deformity [1]. Without sufficient treatment, RA can cause accumulating joint damage, dysfunction, and poor physical functioning, leading to inefficient work capacity, low quality of life, and even death in severe cases [2, 3]. Worldwide, RA is a frequent disease (prevalence of RA is approximately 0.5% and the age-standardized prevalence and incidence rates are increasing), thus rendering this autoimmune and inflammatory disease as a major public health challenge [4]. Therefore, early treatment of RA is crucial. At present, two main medical treatments are considered for RA [5]: disease-modifying antirheumatic drugs (DMARDs) and nonsteroidal anti-inflammatory drugs (NSAIDs). DMARDs, such as methotrexate (MTX), can reduce inflammation, relieve pain, suppress disease activity, and slow cartilage/bone destruction. MTX was the first DMARDs to be approved, and it is currently the most commonly used DMARD for RA patients, as the first-line anchored DMARD to treat RA in clinical settings [6]. However, long-term use of MTX may cause severe systemic complications. In contrast, NSAIDs have been widely used to relieve pain and other inflammatory diseases in clinical settings [7]. However, prolonged use of NSAIDs is linked to the manifestation of several adverse side effects such as gastrointestinal distress, high blood pressure, and cardiovascular complications. In addition to their side effects, both DMARDS and NSAIDs are limited by their high cost. Therefore, it is especially urgent to identify and introduce a novel medicinal plant as an alternative to the current treatment regimes, which will be regarded as both safe and effective on RA.

With over thousands of years of history, Traditional Chinese Medicine (TCM) has been proposed as an alternative therapy to effectually alleviate chronic joint inflammation in arthritis, due to its long medicinal history, strong medical value, mild toxic side effects, and affordable prices [8]. *Aconiti Radix Cocta* (AC) is a dried mother root of *Aconitum carmichaelii* Debx. that was first recorded in the earliest Chinese medicinal classic work, *Shennong's Materia Medica*. AC has been used for the treatment of rheumatism and joint pain, due to its beneficial effects of expelling wind and removing dampness, but also in terms of warming menstruation and relieving pain [9]. AC is the principal herb of Wutou decoction, a representative TCM formula for RA treatment with promising clinical efficacy [10]. Wutou decoction could reverse the abnormal suppression of the peroxisome proliferator-activated receptor-gamma (PPAR-*γ*) coactivator pathway in thermogenesis in collagen-induced arthritis rats with wind-cold-dampness stimulus [11].

The main chemical components of AC are benzoylaconitine, benzoylmesaconine, and benzoylhypacoitine, three major alkaloids with a C19-diterpenoid skeleton. The content range of these principal active ingredients in AC is specified in *The Pharmacopoeia of the People's Republic of China* [12]. All three components have exhibited distinct anti-inflammatory effects on lipopolysaccharide-stimulated macrophages. In particular, benzoylaconitine has shown significant anti-inflammatory activities in *in vivo* experiments, which can reduce primary and secondary paw swelling in AIA rats, and has also demonstrated a therapeutic effect on collagen-induced arthritis [13, 14]. These results indicate that the AC components have a prominent anti-inflammatory potential that can also be used effectively in treating arthritis. However, pharmacological studies on the antiarthritic ability of AC on RA are few. Moreover, the mechanism of reducing inflammation is still unclear. Therefore, it is important to elucidate the mechanism of AC on RA.

In our previous study on protein network [15], the anti-inflammatory action of AC was found to be related to prostaglandins (PGs) synthesis. PGs are metabolites of arachidonic acid via the cyclooxygenase (COX) pathway. COX is a key enzyme that catalyzes the conversion of arachidonic acid to PGs including COX-1 and COX-2 [16]. COX-1 is a structural enzyme that is essential for maintaining human physiological needs [17], whereas COX-2 is an inducible enzyme that is highly expressed during inflammation [18]. COX-2 can enhance PGE2 synthesis during inflammation, which plays a significant role in the subsequent cell inflammatory responses [19], eventually produce a series of inflammatory mediators, and participate in the body's physiological and pathological processes through a cascade reaction. Research has shown that high levels of COX-2 and PGs are detected in synovial fibroblasts obtained from RA patients. Results have confirmed that the increase of PGs content is due to the increased expression of COX-2 [20]. Therefore, we hypothesized that the AC mechanism of action may be related to COX inhibition and thus to the inhibition of the conversion of arachidonic acid to PGs.

The classical complete Freund's adjuvant-induced arthritis (AIA) model resembles human RA damage and is characterized by a rapid onset and progression to polyarticular inflammation. It is a model of chronic inflammation, which is T cell-mediated that shares some features with human RA, including joint swelling, cartilage degradation, loss of joint function, and lymphocyte infiltration of the joints [21, 22]. These properties render this model as suitable for inducing arthritis. Therefore, an AIA rat model was employed to evaluate the effects of AC and its potential pharmacological mechanism on RA treatment.

## 2. Materials and Methods

### 2.1. Preparation of Samples

AC was purchased from Sichuan Neautus Traditional Chinese Medicine Co., Ltd. (Sichuan, China) (Number: D1905110). 50 g AC was soaked in 500 mL pure water for 30 min and boiled for 1 h, gaining the decoction. Then 400 mL pure water was added and boiled for another 1 h, gaining the second decoction. The filtered decoction was combined and concentrated to 50 mL to make 1 g/mL drug stocks and diluted with pure water to proper concentration for *in vivo* study.

Benzoylaconitine, benzoylmesaconine, and benzoylhypacoitine reference standards were dissolved in 0.01% hydrochloric acid methanol and stored at 4°C. The standard solution of benzoylaconitine, benzoylmesaconine, and benzoylhypacoitine was diluted to a concentration gradient of 0.05, 0.1, 0.2, 0.4, 0.8, and 1 mg/mL. All final solutions were filtered through a 0.45 *μ*m filter membrane, and 20 *μ*L filtrate was injected into the HPLC system for analysis.

### 2.2. HPLC-DAD Analysis

Chromatographic separation was performed on a ChromCore PFP column (5 *μ*m, 4.6 × 250 mm) keeping at 30°C. The flow was 1.0 mL/min. The UV wave to determinate benzoylaconitine, benzoylmesaconine, and benzoylhypacoitine was 235 nm. The analysis of mobile phase consisting of acetonitrile (*A*) and 0.04 mol/L ammonium acetate (B) was applied to gradient elute: 0 min, 35% A; 5 min, 40% A; 35 min, 50% A.

### 2.3. Reagents and Antibodies

Complete Freund's adjuvant was supplied from Sigma Company (St Louis, MO, USA). Methotrexate (MTX) was manufactured by SPH Sine Pharmaceutical Laboratories Co., Ltd. (Shanghai, China) (Number: 036180803). Interleukin (IL)-1*β* and IL-17A enzyme-linked immunosorbent assay (ELISA) kits were purchased from Multi Sciences Biotechnology Co., Ltd. (Zhejiang, China). Primary antibodies against COX-1 and COX-2 were supplied from Multi Sciences Biotechnology Co., Ltd. (Zhejiang, China).

### 2.4. Animals

5-6-week male-specific pathogen-free Wistar rats of level weighing 180 ± 20 g were purchased from Chengdu Dashuo Laboratory Experimental Animal Technology Co., Ltd. (Chengdu, China, animal certification number: SCXK (Chuan) 2015-030). The rats were in metal cages with a 12 h : 12 h light-dark cycle in temperature-controlled environment (22 ± 1°C, 55 ± 5% relative humidity) for 1 week before the experiment. All animals and procedures in this study were approved by the Ethics Committee of Chengdu University of Traditional Chinese Medicine.

### 2.5. Clinical Evaluation of AIA Rats

The body weights of rats were gauged at day 7 intervals. The right hind paw volumes (HPV) were assessed via the water displacement method with a plethysmometer on days 0, 7, 14, 21, 28, and 35 until day 42 as primary swelling.

### 2.6. CFA-Induced Chronic Inflammatory Pain

36 rats were divided randomly into 6 groups (*n* = 6): (1) control, (2) AIA (3) MTX, (4) low dose AC (150 mg/kg), (5) middle dose AC (300 mg/kg), and (6) high-dose AC (600 mg/kg) groups. The MTX group was used as the positive control group. The arthritis syndrome of groups (2)–(6) was induced by the intradermal injection with CFA, containing 10 mg heat-inactive BCG in 1 mL paraffin oil, into the right hind paw in 0.1 mL for each rat and followed by a booster immunization on day 28 [23]. The control group received an intradermal injection of 0.1 mL of saline throughout the 42-day experiment.

AIA rats were induced at day 0 and drugs were administered from day 14; then photographic images were taken on day 42, at which point the study was ended. Control group and AIA group rats received normal saline (1 mL/100 g) by oral gavage. Each AC (low, middle, and high) group's rats received AC solution (dose: 150, 300, and 600 mg/kg) by intragastric administration. On day 42, a photo of the right hind paw of the rats was taken; then, all rats were sacrificed after anesthesia. The immune organs and synovial tissues of each rat were collected and weighed. Hind paws samples were collected and then stored at 4% paraformaldehyde.

### 2.7. Indexes of Immune Organs

To assess the effect of AC on immune organs in AIA rats, the indexes of the spleen and thymus were measured. Immune organ index was expressed as the organ weight (mg) versus body weight (g) [24]. Spleen and thymus indexes were calculated according to the following formula:

  Spleen index (mg/g) = spleen weight (mg)/animal body weight (g)
  Thymus index (mg/g) = thymus weight (mg)/animal body weight (g)

### 2.8. Hematoxylin and Eosin (H&E) Staining

The rats were sacrificed on day 42, and the knee joint samples were collected and then stored at 4% paraformaldehyde. The synovial tissue preparation and H&E staining were performed. The slides were examined using the Nikon Eclipse E100 Normal phase microscope (Japan), shooting with Nikon digital sight DS-FI2 imaging system (Japan).

### 2.9. Measurement of IL-1*β* and IL-17A Levels in the Serum

The rats were anesthetized by using 10% chloral hydrate (1 mL/200 g) on day 42. The blood sample was gathered from the retroorbital vein puncture and then centrifuged at 3500 rpm for 15 min to obtain the serum. IL-1*β* and IL-17A in the serum were detected by ELISA kits at 490 nm UV wave in a microplate reader (Thermo, USA).

### 2.10. Western Blotting Assay

After the rats were sacrificed on day 42, the synovial tissues were separated from the knee joints of rats. The expression levels of COX-1 and COX-2 proteins in synovial tissues were assessed using Western blot. Synovial tissue was lysed with ice-cold RIPA buffer, the insoluble materials were removed, and protein concentrations of samples were measured using Protein Assay Kit (Zhejiang, China). The proteins were separated by 10% sodium dodecyl sulfate-polyacrylamide gel electrophoresis and transferred onto polyvinylidene difluoride membranes. After blocking the blots with 5% skimmed milk powder, the membranes were then incubated with anti-COX-1 (1 : 3000, rabbit polyclonal (ab51398-050), Zhejiang, China), anti-COX-2 (1 : 3000, rabbit polyclonal, Zhejiang, China), and anti-*β*-actin (1 : 3000, mouse polyclonal (ab8863-050), Zhejiang, China) overnight at 4°C. Subsequently, the membrane was washed with TBST and incubated with the secondary antibodies at room temperature. The bands were detected by the electrochemiluminescence detection method. The immunoblot intensity was quantified using Alpha software.

### 2.11. RNA Isolation and Quantitative Real-Time PCR (qRT-PCR)

After the rats were sacrificed on day 42, the synovial tissues were separated from the knee joints of rats. The gene expression levels of COX-1 and COX-2 in synovial tissues were assessed using qRT-PCR. RNA in synovial tissue was isolated using the TriPure Isolation Reagent. The first strand cDNA was reversely transcribed and used as the DNA templates for analysis of gene mRNA relative expression levels. qRT-PCR reactions were conducted on a CFX96 Real-Time PCR system with the Transcriptor First Strand cDNA Synthesis Kit (GHA). PCR reaction was set as follows: 95°C for 2 min, 95°C for 10 s, 40 cycles of 60°C for 30 s, and 95°C for 15 s. It was performed in triplicate for each sample and the relative expression level was expressed using 2^−△△Ct^ method (Table 1).

### 2.12. Statistical Analysis

All statistical analyses were calculated by using one-way ANOVA followed by the post hoc Dunnett's test, which were applied to evaluate the significant differences among the groups. Data were presented as mean ± standard deviation (SD) and *P* < 0.05 was considered to be statistically significant. All data were analyzed by the SPSS 17.0 software.

## 3. Results and Discussion

### 3.1. HPLC-DAD Analysis of AC

**Figure 1:**
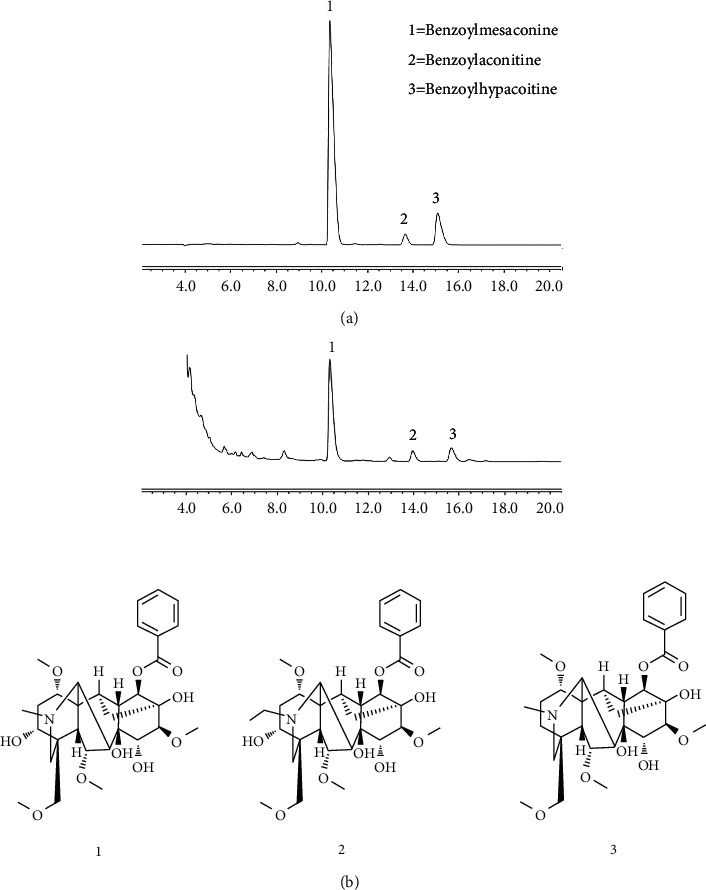
Chromatograms of (a) reference standards and (b) AC decoction.

Three chemicals were quantified by calculating the peak area, and they were identified by comparing the retention times of each peak with the authentic references. The contents of the three main chemicals were found to be 0.084% for benzoylaconitine, 0.0276% for benzoylmesaconine, and 0.004% for benzoylhypacoitine (Figure 1).

### 3.2. Clinical Evaluation of AIA Rats

**Figure 2:**
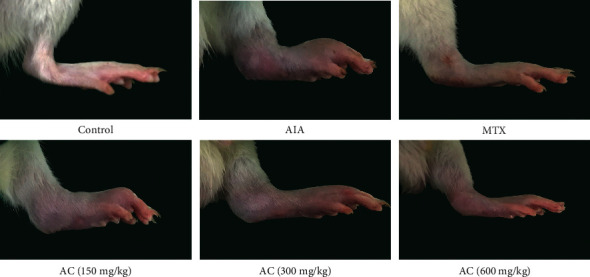
Representative photos of paw swelling of each group on the 42nd day.

As shown in Figure 2, the redness and swelling of the right hind paw volume (HPV) of rats in the AIA group were markedly increased compared with the rats in the control group. Following AC treatment with 300 and 600 mg/kg since day 14, paw swellings were then markedly reduced compared to the AIA group.

**Figure 3:**
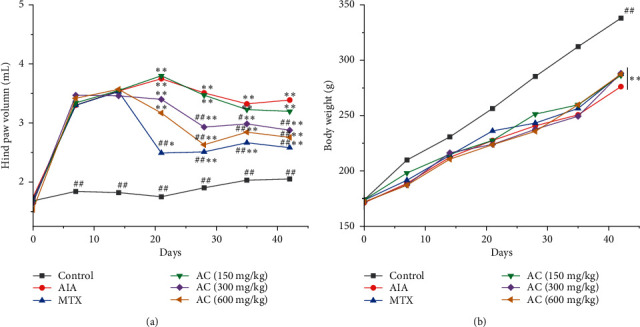
(a) Hind paw volume during 42 days between the AIA and the AC treatment. (b) Effect of AC on body weight. ^*∗*^*P* < 0.05 and ^*∗∗*^*P* < 0.01, compared with the control group; ^#^*P* < 0.05 and ^##^*P* < 0.01, compared with the AIA group (*n* = 6).

HPV at days 0, 7, 14, 21, 28, 35, and 42 was evaluated in terms of swelling severity by complete Freund's adjuvant- (CFA-) induced. As shown in Figure 3(a), the HPV of the model group initially increased and reached its maximum value on day 21. Then, it decreased slowly from day 21 to 28. After booster immunization on day 28, the HPV slowly started to increase from day 35. The entire paw swelling process in the AIA group emulated the state of arthritis. Figure 3(a) shows that the HPV of the AIA group was markedly increased compared with the control group. In contrast, the swelling of rats in the treatment groups was mitigated, and AC could alleviate paw swelling from day 14. On day 21, a slight decrease in AC (600 mg/kg) was observed compared with the AIA group. Between days 28 and 42, rats were treated with 300 mg/kg and 600 mg/kg of AC (*P* < 0.01), and they exhibited a significantly lower HPV compared to AIA rats, which were dose-dependent.  Figure 3(b) shows the results of body weight, which was used to evaluate AC improvement on the holistic health of AIA rats. Compared with the control group, all other groups exhibited a significant loss in their body weight.

### 3.3. Effects of AC on the Immune Organ Index

**Figure 4:**
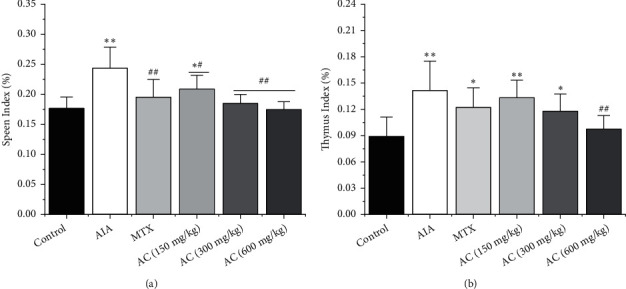
Immune organ index in each group. (a) Indexes of the spleen. (b) Indexes of the thymus. ^*∗*^*P* < 0.05 and ^*∗∗*^*P* < 0.01, compared with the control group; ^#^*P* < 0.05 and ^##^*P* < 0.01, compared with the AIA group (*n* = 6).

Spleen and thymus are important immune organs, and the organ index can reflect the strength of the immune function. The immune organ index is shown in Figures 4(a) and 4(b). Compared with the control group, CFA-induced rats showed significant enlargement on spleen and thymus tissues. After treatment with AC, the spleen index of the AC (150, 300, and 600 mg/kg) group was significantly reduced compared with the AIA group. Similarly, the thymus index of the AC (600 mg/kg) group also showed a signiﬁcant reduction compared with the AIA group.

### 3.4. AC Restores Histological Changes of the Synovial Tissue in AIA Rats

**Figure 5:**
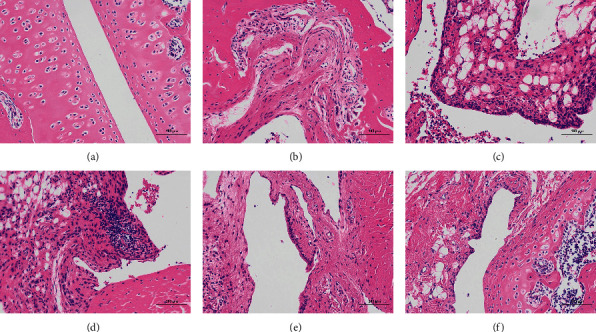
Histopathology of the right hind knee joint in each group (x 200, scale bars = 100 *μ*m). (a) Control; (b) AIA; (c) MTX; (d) AC (150 mg/kg); (e) AC (300 mg/kg); (f) AC (600 mg/kg).

The effects of AC on cartilage damage and synovial hyperplasia in the knee joint of AIA rats were evaluated by histopathological studies. As shown in Figure 5, the knee joints in the AIA group showed severe synovial tissue proliferation, inflammatory cell infiltration, and cartilage and bone erosion compared to the control group. The AC (150 mg/kg) group revealed significant inflammatory cells infiltration in the synovial membrane. In contrast, mild inflammatory cells infiltration was observed in the MTX and AC (300 and 600 mg/kg) groups, which also demonstrated normal histological structure. This finding suggests that MTX and AC (300 and 600 mg/kg) could reverse CFA-induced histopathological alterations in the synovial membrane, articular cartilaginous surface, and bony structure.

### 3.5. Effects of AC on IL-1*β* and IL-17A Levels

**Figure 6:**
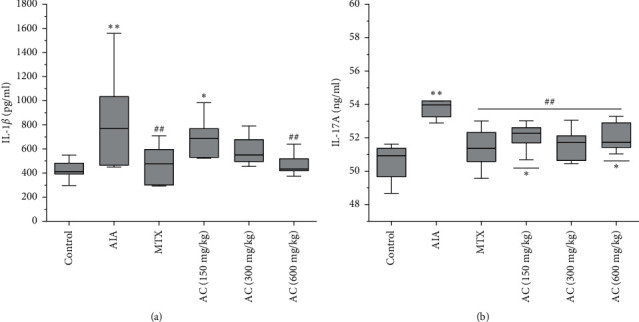
Effects of AC on IL-1*β* and IL-17A levels in serum via ELISA. ^*∗*^*P* < 0.05 and ^*∗∗*^*P* < 0.01, compared with the control group; ^#^*P* < 0.05 and ^##^*P* < 0.01, compared with the AIA group (*n* = 6).

As shown in Figures 6(a) and 6(b), IL-1*β* and IL-17A were measured in order to assess the anti-inflammation effect of AC. IL-1*β* and IL-17A levels were found to be significantly increased in the AIA group compared to the control group (*P* < 0.01). The expressions of IL-1*β* in the AC (600 mg/kg) and MTX group were significantly reduced compared to the AIA group. IL-17A production after AC (150, 300, and 600 mg/kg) and MTX group administration were also markedly reduced compared to the AIA group. Therefore, AC can obviously inhibit inﬂammatory cytokines in the serum of AIA rats.

### 3.6. Effects of AC on COX-1 and COX-2 Protein Expression

**Figure 7:**
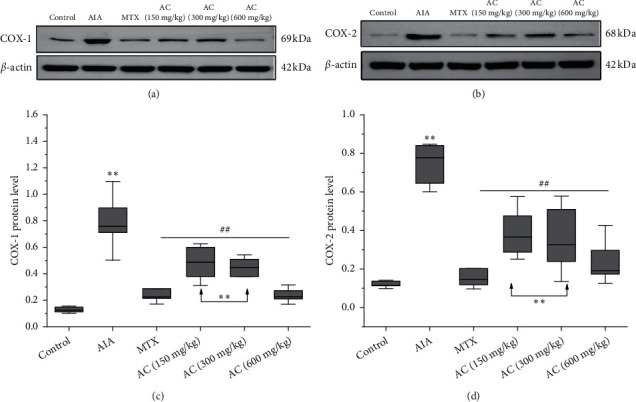
(a) Protein expression of COX-1 in the synovial tissues. (b) Protein expression of COX-2 in the synovial tissues. (c) Western blot analysis of the expressed level of COX-1 in the synovial tissues. (d) Western blot analysis of the expressed level of COX-2 in the synovial tissues. ^*∗*^*P* < 0.05 and ^*∗∗*^*P* < 0.01, compared with the control group. ^#^*P* < 0.05 and ^##^*P* < 0.01, compared with the AIA group (*n* = 6).

To further investigate the anti-inflammatory mechanism of AC on CFA-induced adjuvant arthritis in rats, we detected the inflammation-related signaling pathway of cyclooxygenase. As shown in Figure 7, the AIA group showed significant elevation in both COX-1 and COX-2 levels compared with the control group (*P* < 0.01), whereas AC could effectively downregulate the protein levels of COX-1 and COX-2. The AC (300 and 600 mg/kg) groups revealed a significant decrease in COX-1 and COX-2 compared with the AIA group (*P* < 0.05; *P* < 0.01). Among the three doses of AC groups, 600 mg/kg exhibited the strongest effect (*P* < 0.01), and there was no significant difference compared with the control group.

### 3.7. Effects of AC on COX-1 and COX-2 Gene Expression

**Figure 8:**
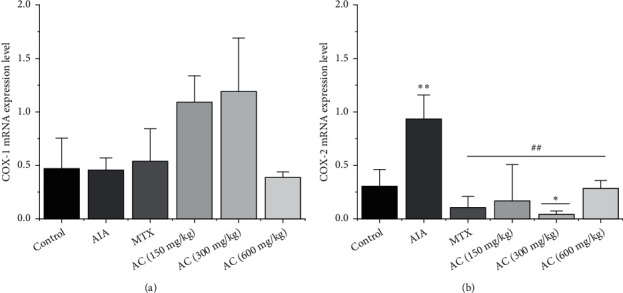
(a) qRT-PCR analysis of the expressed level was presented of COX-1 in the synovial tissues; (b) qRT-PCR analysis of the expressed level was presented of COX-2 in the synovial tissues. ^*∗*^*P* < 0.05 and ^*∗∗*^*P* < 0.01, compared with the control group. ^#^*P* < 0.05 and ^##^*P* < 0.01, compared with the AIA group (*n* = 6).

To further substantiate the above findings, we measured the mRNA levels of COX-1 (Figure 8(a)) and COX-2 (Figure 8(b)) in synovial tissues by real-time quantitative reverse transcription polymerase chain reaction (qRT-PCR) to evaluate the effects of AC at the transcriptional level, and the respective findings are shown in Figures 8(a) and 8(b). COX-1 mRNA expression in the AIA group rats remained unchanged compared to the control group (*P* > 0.05). A tendency of elevated COX-1 mRNA levels by the AC (150 and 300 mg/kg) groups was found, which was nonsignificant. Although the AC (600 mg/kg) group can reduce the COX-1 mRNA levels, it did not reveal statistically significant differences with the control group and AIA group (*P* > 0.05). Furthermore, COX-2 mRNA was significantly upregulated in the AIA group compared with the control group (*P* < 0.01). The mRNA levels of COX-2 in the AC (150, 300, and 600 mg/kg)-treated groups were significantly reduced compared with the AIA group (*P* < 0.01). According to the qRT-PCR results, the AC (150, 300, and 600 mg/kg) groups exhibited clear effects on reducing the mRNA levels of COX-2 in synovial tissues. However, the AC group did not induce any significant effect on COX-1 mRNA expression in RA rats' synovial tissues.

## 4. Discussion

AIA is a commonly used method to assess the antiarthritic effect of various agents, with many of the clinical and pathological characteristics found in human RA condition. We employed the AIA rat model to evaluate the antiarthritic activity of AC. Intradermal injection of CFA could induce infiltration of the synovial membrane, production of proinflammatory factors, and accumulation of neutrophils, resulting in persistent nociceptive stimulation. AIA induced significant inflammatory (lymphocytes) cell infiltration, synovial hyperplasia, cartilage erosion, and pannus formation, as shown in the respective histopathological images. Additionally, AIA significantly increased the levels of IL-1*β*, IL-17A in serum. Chronic inflammation in the AIA model was mainly manifested as a progressive increase in the volume of the injected paw. Moreover, the arthritic rats lost weight compared with the control group. These results demonstrate the success of the arthritis model employed.

The metabolic and immunomodulatory activity of different drugs is typically evaluated based on both body weight and immune organ index values in treated animals [25]. CFA induced can lead to profound immunosuppression and to a reduction in rat body weight caused by promoting the production of macrophages and lymphocytes [26]. Spleen and thymus weights relate to the number of immune cells, and they both play an important role in cellular immunity by serving as reservoirs for antibody storage [27]. In this study, the spleen and thymus indexes of the AC (300 and 600 mg/kg) groups signiﬁcantly decreased, which is possibly caused by a reduction in AC lymphocyte proliferation. The results of the organ index showed that AC could enhance the body's immune function.

MTX could greatly alleviate the swelling of the rats' hind paw in the first 14 days, thus indicating a quick effect. This may be related to the direct anti-inflammatory effect and inhibition of leukocyte tropism of MTX. However, MTX exerted its weakened pharmacological effect after booster immunization, when, at the same time, AC gradually exerted its beneficial effects. AC was found to greatly inhibit the development of the disease. This suggests that AC has a lasting therapeutic effect on RA. At the initial stage of AC administration, a slight reduction in the paw swelling of rats was observed, which accumulated with the administration time. AC gradually exerted its efficacy at a later stage, and its efficacy was equal to the efficacy of MTX. Therefore, we speculate that the efficacy of AC may be attributed to a cumulative effect.

RA is characterized by persistent inflammatory synovitis. Proinflammatory cytokines play a vital role in the pathogenesis of synovitis and articular destruction in RA. Thus, they are both important targets for RA treatment [28, 29]. Interleukin-1*β* (IL-1*β*) and interleukin-17A (IL-17A), as proinflammatory cytokines, have been reported to be significantly expressed in the rheumatoid joint [30], which could lead to joint synovial destruction. IL-1*β*, the predominant extracellular form of IL-1, has been regarded as an important proinflammatory cytokine in the pathogenesis of RA. It is produced at the early stages of the immune response, with a strong proinflammatory effect, which mainly causes bone and cartilage destruction [31]. IL-17A also plays a crucial role in RA. It is mainly produced by T cells and leads to osteoclasts activation and to the increased production of other cytokines [32, 33]. In the AIA model, activation and release of proinflammatory cytokines and interleukins were found to alter osteoclast differentiation, leading to bone erosion [34]. Histopathological images of synovial tissues revealed the pronounced cell infiltration of lymphocytes accompanied by bone erosion and angiogenesis. In this study, AC reduced the concentrations of inflammatory cytokines IL-1*β* and IL-17A. In addition, AC not only suppressed inflammatory responses but also alleviated bone erosion and neovascularization in AIA rats. Consequently, AC may be a promising candidate drug for RA treatment.

Cyclooxygenase isoenzymes (COX-1 and COX-2) have a critical role in inflammation through arachidonic acid metabolism pathways. COX-1 is constitutively expressed in the majority of normal tissues for maintaining normal physiological effects, whereas COX-2 is specifically expressed in inflamed tissues and it is unregulated [35, 36]. COX-2 participates in the generation of proinflammatory factors and cartilage destruction, which plays a major role in inflammation and autoimmune disease [37]. Crofford et al. [38] reported that IL-1*β* could enhance COX-2, mRNA, and protein synthesis in rheumatoid synovial cells but had no effect on COX-1. Early studies had stated that the severe gastrointestinal toxicity of NSAID is due to its inhibitory effect on COX-1 [39]. However, a recent research has shown that selective inhibition of COX-1 activity can effectively treat inflammation without causing gastric damage and cardiovascular disease [40]. Therefore, it is essential to conduct further studies to elucidate whether inhibition of COX-1 can treat RA.

In this study, the effects of AC on COX-1, COX-2 mRNA, and protein levels in AIA rat synovial tissues were also investigated. Our findings suggest that COX-1 and COX-2 expression were significantly suppressed by AC in translation level. The results suggest that inhibition of COX catalyzed the conversion of arachidonic acid into prostaglandins, and this may be the mechanism by which AC mediates its anti-inflammatory activity. However, we found that COX-1 mRNA level and protein expression are inconsistent. This may be due to the fact that there are three mRNA transcripts in cyclooxygenase: 2.8 kb, 4.5 kb, and 5.2 kb. The 2.8 kb transcript is the most frequently detected because it probably encodes the primary protein form for COX-1 [41]. The COX-1 mRNA level that we detected and which may represent the total mRNA level remained unchanged in the AIA group. However, there may also be additional factors that play a role in this process, such as the effect of posttranslational modification or the fact that the protein was changed from an inactive to an active state by an activating factor. There was no positive correlation between this change and the change in the COX-1 transcription level. The levels of COX-1 mRNA in the synovial tissue of the AIA rats remained unchanged, whereas the level of COX-1 protein increased, which may reveal that regulation of translation level may play a leading role in this process. We also found that the COX-1 mRNA level in the AIA group was not significantly different compared to the control group. However, the mRNA level of COX-1 was increased in the AC (150 mg/kg and 300 mg/kg) groups. Although it is generally known that COX-1 limits inflammatory responses, mild modifications can occur in the expression of the enzyme in cells and tissues when they are stimulated by proinflammatory cytokines or injury [42]. Therefore, the regulatory effect of RA on AC in COX-1 transcription level needs further study.

## 5. Conclusion

In summary, our study indicates that AC is significantly against adjuvant-induced arthritis in rats, by inhibiting immune organs (spleen and thymus), attenuating paw swelling, infiltration of inflammatory cells and synovial hyperplasia, and downregulating the levels of IL-1*β* and IL-17A in AIA rats. In addition, we identified that AC has significant anti-inflammatory therapeutic effects. Further molecular mechanism is to regulate arachidonic acid metabolism pathways. Particularly, AC could ameliorate overexpression of COX-2. Although these results show that the use of AC for RA treatment can be very promising, further research should be carried out to elucidate in greater detail the molecular mechanisms involved in these antiarthritic properties of AC.

## Figures and Tables

**Table 1:** Primer pair sequences used for qRT-PCR.

Genes	Forward	Reverse
COX-1	CAGGAGGTGTTTGGGTTGCT	CTATAAGGATGAGGCGAGTGGT
COX-2	ACTACGCCTGAGTTTCTGACAAG	TTCACAATGTTCCAGACTCCCT
*β*-Actin	TGTCACCAACTGGGACGATA	GGGGTGTTGAAGGTCTCAAA

## Data Availability

The data used to support the findings of this study are included within the article.
